# Culturally and linguistically diverse voices and views in COVID-19 pandemic plans and policies

**DOI:** 10.5365/wpsar.2022.13.2.915

**Published:** 2022-05-02

**Authors:** Nafiseh Ghafournia, Peter D Massey, Sunita J Rebecca Healey, Bhavi Ravindran

**Affiliations:** a Multicultural Health Service, Hunter New England Health, Wallsend, New South Wales, Australia.; b The University of Newcastle, Callaghan, New South Wales, Australia.; c College of Medicine and Dentistry, James Cook University, Townsville, Queensland, Australia.

## Abstract

**Objective:**

This paper presents a rapid assessment of coronavirus disease 2019 (COVID-19) pandemic plans and explores the representation of culturally and linguistically diverse (CALD) communities in such plans. Four levels of pandemic plans were reviewed: regional, state, national and international.

**Methods:**

Discussions with representatives from four CALD communities informed the development of search and selection criteria for the COVID-19 plans, which were gathered and assessed using a CALD lens. Six COVID-19 pandemic plans that met the inclusion criteria were critically assessed.

**Results:**

The reviewed plans did not report any CALD community voices, views or consultations with community groups in the development phase, nor did they acknowledge the diversity of CALD populations. A few plans noted the vulnerability of CALD communities, but none discussed the challenges CALD communities face in accessing health information or health services during the pandemic, or other structural barriers (social determinants of health).

**Discussion:**

Our analysis revealed major gaps in all pandemic plans in terms of engaging with immigrant or CALD communities. Policies and plans that address and consider the complex needs and challenges of CALD communities are essential. Collaboration between public health services, multicultural services and policy-makers is vital for the inclusion of this higher-risk population.

In Australia, as in other developed nations, the coronavirus disease 2019 (COVID-19) pandemic has disproportionately affected culturally and linguistically diverse (CALD) communities. In many countries, immigrant communities have experienced higher rates of COVID-19 infection, hospitalization, severity of disease and death. (*1*-*3*) We know that, in Australia, the risk of transmission and serious illness from COVID-19 is not equal across the population – one vulnerable population disproportionately impacted by COVID-19 is people from CALD backgrounds. (*4*)

The discrepancies in outcomes between CALD and non-CALD populations evident through the COVID-19 pandemic were also seen during the 2009 H1N1 pandemic. (*5*, *6*) It has long been argued that the principles of social justice and corrective justice must be applied in pandemic planning, to enable risk reduction in populations where the need is greatest. (*7*)

Families and communities are the ultimate recipients of the effects of pandemic plans, and thus need to be involved in their development. Any pandemic health policy or plan must address the public’s real concerns and needs, especially among groups who are at higher risk, because this will lead to a reduction in risk for the whole population. (*8*, *9*) Not engaging with vulnerable communities when developing health policies or plans is not only unfair but also endangers the health of the broader population. (*9*)

This article presents a rapid assessment of COVID-19 pandemic plans applicable to the region of the public health unit (PHU) conducting the study. It explores whether the needs, expectations and challenges of CALD communities are represented in these plans. The terms “immigrant” and “CALD communities” are used interchangeably here. The study did not include a review of all relevant literature or research papers; rather, the focus was on pandemic plans and policies at multiple settings and levels (from regional to international).

## METHODS

Pandemic plans at regional, state, national and international levels were selected, accessed and then critically assessed through a “CALD lens.”

The selection criteria included plans that were:

available online at the time of assessment;
updated or published within the past 5 years; and
applicable to the region or state of the PHU undertaking the review, to a neighbouring state or to international plans published by the World Health Organization (WHO).

As part of using a CALD lens, discussions with representatives from four CALD communities informed the development of the search and selection criteria. These key informants were e-mailed a series of questions. Four consumer representatives of local multicultural health services then discussed the e-mailed questions with the informants to finalize the assessment questions. The final questions were as follows:

Does the plan describe a governance structure that includes CALD community representatives?
Does the plan describe any consultation with CALD communities before or during the development of the plan?
Does the plan outline how it reflects and embraces the diversity of CALD communities?
Does the plan reference the challenges CALD communities encounter in accessing health systems?
Does the plan describe how CALD communities would be involved in the oversight, implementation or review of the plan when it is operationalized?

## Results

Six plans met the inclusion criteria and were critically reviewed (**Table 1**). All were found to have major gaps in terms of engaging with immigrant or CALD communities and addressing real health needs and challenges. None of the reviewed plans reported any voices, views or consultations with CALD community groups in the development phase. There was some mention of the significance of community engagement in the process of policy-making, but there were no details on which communities or how to engage with them.

**Table 1:** Pandemic plans reviewed by setting

Plan	Setting	Web site
Hunter New England Pandemic Plan for Influenza and Other Respiratory Infections	Regional	https://www.hnehealth.nsw.gov.au, accessed 31 August 2020
NSW Health Influenza Pandemic Plan	State	https://www1.health.nsw.gov.au/pds/ActivePDSDocuments/PD2016_016.pdf, accessed 31 August 2020
COVID-19 Pandemic Plan for the Victorian Health Sector	State	Link is no longer active; available from the corresponding author upon request
Australian Health Sector Emergency Response Plan for Novel Coronavirus (COVID-19)	National	https://www.health.gov.au/resources/publications/australian-health-sector-emergency-response-plan-for-novel-coronavirus-covid-19, accessed 31 August 2020
COVID-19 strategy update, World Health Organization	International	https://www.who.int/publications/m/item/covid-19-strategy-update, accessed 31 August 2020
Operational planning guidelines to support country preparedness and response, COVID-19 strategic preparedness and response plan, World Health Organization	International	https://www.who.int/publications/i/item/draft-operational-planning-guidance-for-un-country-teams, accessed 31 August 2020

The assessed plans included no acknowledgement of the diversity of CALD populations. When immigrant or CALD communities were named, they were presented incorrectly as generalized and homogeneous communities.

The vulnerability of immigrant and refugee or CALD communities was noted in three plans (the two WHO plans and the COVID-19 Pandemic Plan for the Victorian Health Sector). However, none of the documents discussed CALD community challenges in accessing health information or health services during the pandemic or other structural barriers such as social determinants of health. Also, the documents did not mention factors such as unemployment, crowded housing, visa status, low health literacy, racism and cultural beliefs. A few references were made to challenges in communication between health organizations and immigrant communities, but the plans tended to fall short of addressing solutions for overcoming barriers to reduce risk. Only two plans (the NSW Health Influenza Pandemic Plan and the COVID-19 Pandemic Plan for the Victorian Health Sector) talked about the necessity of providing translated information.

## Discussion

In general, Australian policies and plans do not engage with CALD communities, and there is little data regarding the needs of these communities and the challenges they face in accessing health-care systems. This may be an indication of structural racism in the system. (*10*) Policies and plans that address and consider the complex needs of and challenges faced by CALD communities are essential, (*8*, *9*) and their development must include the knowledge and expertise of diverse groups from CALD communities and multicultural service providers. We call for health plans and policies to be redeveloped to be inclusive, culturally responsive and based on consultation with CALD communities. There must be a clear process of engagement, respectful and meaningful two-way communication between policy-makers and CALD communities to identify culturally appropriate and effective public health control strategies.

## Conclusion

Despite the health inequities faced by people from CALD communities, their voices and needs were not reflected in the pandemic plans assessed in this study. The plans failed to address embedded inequities, which are particularly important in health emergencies. It is recommended that CALD communities be included in the development and implementation of pandemic plans. Further research should be undertaken with diverse communities to enable effective public health actions for COVID-19 and future pandemics.
